# CAD systems for colorectal cancer from WSI are still not ready for clinical acceptance

**DOI:** 10.1038/s41598-021-93746-z

**Published:** 2021-07-13

**Authors:** Sara P. Oliveira, Pedro C. Neto, João Fraga, Diana Montezuma, Ana Monteiro, João Monteiro, Liliana Ribeiro, Sofia Gonçalves, Isabel M. Pinto, Jaime S. Cardoso

**Affiliations:** 1 INESCTEC, 4200-465 Porto, Portugal; 2 Faculty of Engineering (FEUP), University of Porto, 4200-465 Porto, Portugal; 3 IMP Diagnostics, 4150-146 Porto, Portugal; 4 ICBAS, University of Porto, 4050-313 Porto , Portugal; 5 Cancer Biology and Epigenetics Group, IPO-Porto, 4200-072 Porto, Portugal

**Keywords:** Gastrointestinal cancer, Colorectal cancer, Data processing, Image processing, Computational biology and bioinformatics, Machine learning, Gastrointestinal cancer, Colorectal cancer, Diagnosis, Pathology, Biomedical engineering

## Abstract

Most oncological cases can be detected by imaging techniques, but diagnosis is based on pathological assessment of tissue samples. In recent years, the pathology field has evolved to a digital era where tissue samples are digitised and evaluated on screen. As a result, digital pathology opened up many research opportunities, allowing the development of more advanced image processing techniques, as well as artificial intelligence (AI) methodologies. Nevertheless, despite colorectal cancer (CRC) being the second deadliest cancer type worldwide, with increasing incidence rates, the application of AI for CRC diagnosis, particularly on whole-slide images (WSI), is still a young field. In this review, we analyse some relevant works published on this particular task and highlight the limitations that hinder the application of these works in clinical practice. We also empirically investigate the feasibility of using weakly annotated datasets to support the development of computer-aided diagnosis systems for CRC from WSI. Our study underscores the need for large datasets in this field and the use of an appropriate learning methodology to gain the most benefit from partially annotated datasets. The CRC WSI dataset used in this study, containing 1,133 colorectal biopsy and polypectomy samples, is available upon reasonable request.

## Introduction

**Figure 1:**
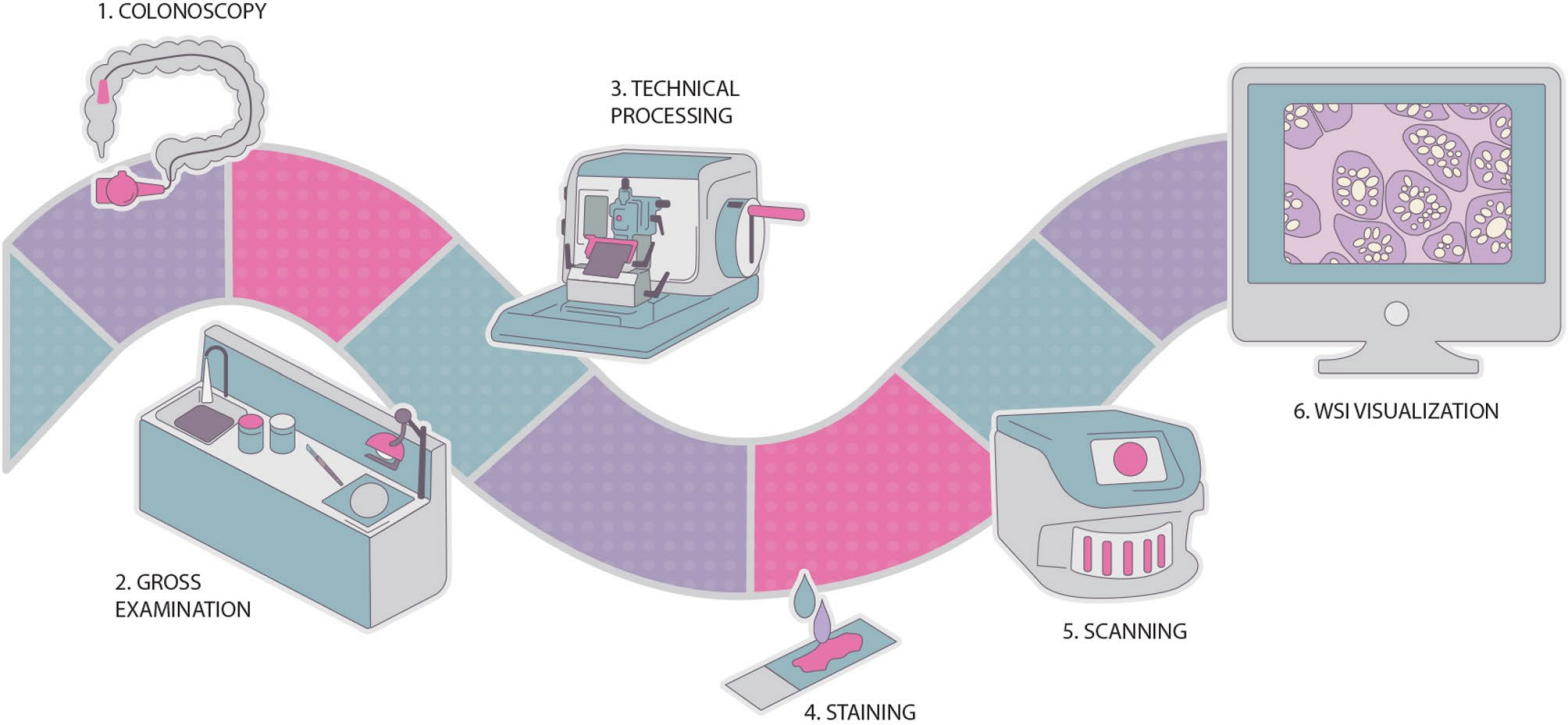
Digital pathology workflow, from collecting the biopsy sample to the WSI visualisation.

Pathologists are responsible for the diagnosis of samples collected during biopsies. Digital pathology methods have been increasing due to technological advances, and their implementation can support the work conducted by pathologists. And while this multi step process requires an additional scanning step (Fig. 1), the benefits far outweigh the increased initial overhead of these steps. For example, access to old cases, collaboration with external laboratories, and data sharing are all made easier. For example, peer-review of a whole-slide image (WSI) is completed at a quicker pace with a digital pathology workflow. In addition, the ability to easily access images mitigates the risk of errors, making diagnosis more auditable.

Over the last decade, the advent of digitised tissue samples, the wider adoption of digital workflows in pathology labs, and the consequent availability of more data, combined with a shortage of pathologists, enabled the evolution of the computational pathology field with the integration of automatic image analysis into clinical practice, mainly based in Artificial Intelligence (AI) methodologies^1–4^. Researchers have been exploring the implementation of computer-aided diagnosis (CAD) systems for several different tasks regarding cancer WSI. The most popular are the detection, grading and/or segmentation of lesions. Additionally, there are also predictive systems that attempt to estimate the patient’s probability of survival.

Despite the ever-growing number of publications of machine learning (ML) methods applied to CAD systems, there is a dearth of published work for the task of joint detection and classification of colorectal lesions from WSI, lagging colorectal cancer (CRC) behind pathologies such as breast cancer and prostate cancer. Furthermore, a significant amount of the work developed does not use the entire WSI but instead uses crops and regions of interest extracted from these images. While these latter works show significant results, the applicability of such works in clinical practice is limited. Similarly, publicly available datasets often consist of crops instead of the original image. Others include only abnormal tissue, limiting the development of CRC diagnostic systems and the detection task.

As a first step in addressing these limitations, in this work, we examine and identify the benefits and shortcomings of the current body of work on CAD systems for CRC diagnosis from WSI. Since the development of such systems typically requires large and diverse datasets, any review would be incomplete without a concurrent discussion of the existing data and associated annotation metadata. Following this initial reflection, and in an attempt to better understand the limitations of the data and methods used to develop CAD systems for CRC diagnosis, we analyse the impact of using partially annotated datasets, as well as the effect of increasing training instances, on the robustness of learning. We conduct this empirical study using in-house data, an ongoing effort to develop a reference dataset for CRC automatic diagnosis. These comparisons serve two distinct purposes: First, they validate the annotation and labelling efforts used in the dataset construction; Second, public data is used to evaluate the dependency of the performance with the number of used samples. One method was assessed for its applicability within a framework for pathological diagnosis in a digital workflow. In an attempt to address the lack of public CRC datasets with large numbers of slides, the colorectal data used in this study, that sums up 1,133 colorectal biopsy and polypectomy samples, is available upon reasonable request.

This paper distinguish itself from previous reviews^5,6^ by focusing only on CRC histopathological slides classification, distinguishing works that evaluate complete slides from those that use only smaller portions of the image, without returning a final diagnosis for each case. Moreover, we discuss their major shortcomings, especially with regard to the properties of datasets that are used. In this review, we provide a detailed description of the proposed methods, including most of the references of the previous reviews and, in addition, some more recent works, making this a timely paper.

In addition to this introductory section, this paper consists of three other main sections and a conclusion. The development of AI models for medical applications should always take into consideration the clinical background, and thus “Clinical insights” section introduces the main clinical notions regarding CRC and overviews the process of classifying lesions. Afterwards, “Computational pathology” section provides a detailed review of CAD systems for CRC diagnosis from WSI, discussing the current problems regarding the analysis of these images. Finally, before the conclusion and discussion of future work on “Conclusions” section, “Feasibility study on the use of weakly annotated and larger datasets for CRC diagnosis” section not only describes the workflow of the CRC dataset construction, but also shows the results of our feasibility study on the use of weakly annotated and larger datasets.

## Clinical insights

### CRC epidemiology

Colorectal cancer (CRC) represents one of the major public health problems today. A striking and often unknown fact is that CRC is the second most deadly cancer^7,8^. Globocan estimated data for 2020 show that CRC is the third most incident cancer (10% of all cancers) and the second most deadly (9,4%; surpassed by lung cancer, 18%)^8^. CRC is the third most common cancer in men (after lung and prostate cancer) and the second most common cancer in women (after breast cancer). CRC is a disease of modern times: the highest rates of incidence happen in developed countries^9^. As the world becomes richer, and people shift to a western lifestyle, the incidence of CRC is expected to increase, since it is a multifactorial disease resulting from lifestyle, genetic, and environmental factors^9,10^. Population growth and ageing lead to an increasing incidence of the disease, as well as better and more numerous screening programs for early detection and prevention. The prevalence of screening among individuals aged 50 years and older increased from 38%, in 2000, to 66%, in 2018, according to data from the National Center for Health Statistics (NHIS)^11^. Importantly, CRC is a preventable and curable cancer if detected early on, and, therefore, screening is an effective tumour prevention measure^12^. Screening determines the decrease in mortality through timely detection and removal of adenomatous polypoid (pre-malignant) lesions, promoting the interruption of progression to cancer. It should begin with colonoscopy in asymptomatic individuals aged 50 years or over (and without personal or family risk factors for CRC) and repeated every ten years if normal^13^. It is worth mentioning that, due to the Covid-19 pandemic, CRC screening programmes have been disrupted worldwide. As such, it is crucial that catch-up screening is provided as soon and effectively as possible, hoping to mitigate the impact on CRC deaths^14,15^. Computer-aided diagnosis (CAD) solutions in CRC could help in this task, contributing to improve pathology diagnostic capacity.

### CRC pathological assessment

**Figure 2:**
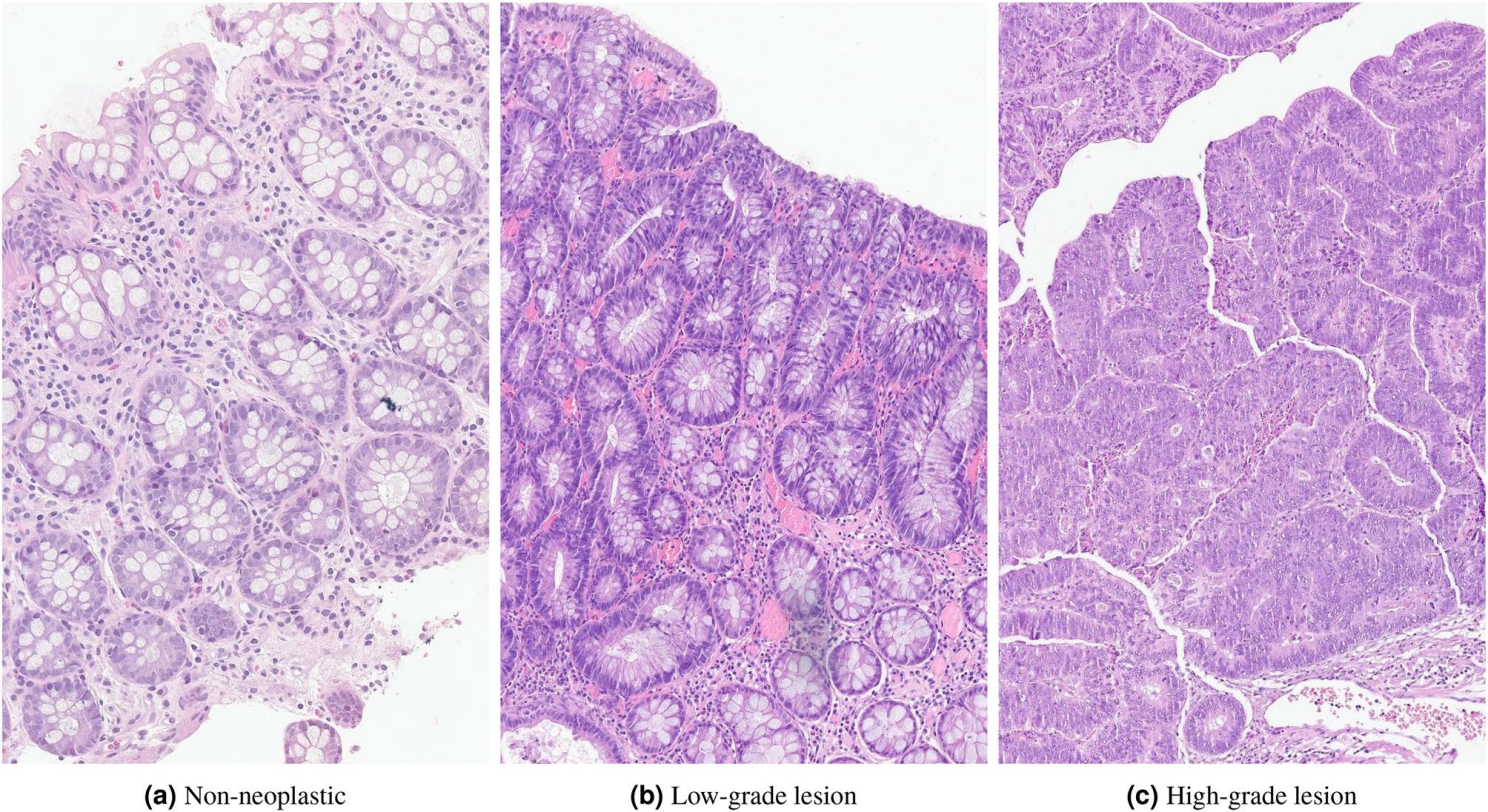
Normal colonic mucosa and dysplastic progression.

During pathological assessment, colorectal biopsies/polyps can be stratified in non-neoplastic, low-grade dysplasia (LGD), high-grade dysplasia (HGD, including intramucosal carcinomas) and invasive carcinomas, regarding their development sequence. Colorectal dysplasia refers to the pre-malignant abnormal development of cells/tissues, which can eventually progress to tumour lesions, and is classified in low- and high-grade, with the last confering a relative higher risk of cancer (Fig. 2).

#### The case for grading dysplasia

It is well-known that grading colorectal dysplasia is a somewhat subjective issue. In a study to evaluate inter-observer variability in HGD diagnosis, five gastrointestinal pathologists conducted a consensus conference in which criteria for colorectal HGD were developed^16^. When grading the same 107 polyps, the inter-observer agreement was found to be poor both before and after the consensus. Other studies have also shown sub-optimal agreement in grading colorectal dysplasia^17,18^. Despite this, the most recent guidelines from the European Society of Gastrointestinal Endoscopy (ESGE), as well as those from the US multi-society task force on CRC, continue to recommend surveillance for polyps with high-grade dysplasia regardless of their size^13,19^. Patients requiring surveillance after polypectomy include those with complete removal of:

- at least one adenoma $\ge ~10~mm$ or with high-grade dysplasia;
- five or more adenomas;
- any serrated polyp $\ge 10~mm$ or with dysplasia^13^.

As such, it remains a current practice in most countries (although not in every laboratory) to evaluate and grade colorectal dysplasia.

#### Dysplasia grading

As previously stated, various studies have shown good concordance among pathologists in recognising adenomatous features, but lower levels of agreement evaluating dysplasia grade, with significant inter-observer variability^16–18^. To date, there are still no tangible criteria on what distinguishes the high end of LGD from the low end of HGD. Although there are some reporting guidelines regarding grading dysplasia in colorectal biopsies^20–22^, objective criteria are still lacking. It is fairly easy for a pathologist to diagnose a typical low grade or high-grade adenoma but, since in fact these lesions exist in a continuum, the correct assessment of many intermediate cases is more difficult. Nevertheless, protocols such as the English National Health System (NHS) bowel cancer screening programme guidance, with guidelines from the bowel cancer screening programme pathology group^20^ or the Pan-Canadian consensus for colorectal polyps report^22^, can aid pathologists to grade colorectal lesions more objectively. Additional information from reference books, such as the World Health Organization (WHO) Classification of Tumours: digestive system tumours^7^, can also assist in this task. The most relevant characteristics that differentiate low- and high-grade dysplasia are detailed in Table 1.

**Table 1:** Colorectal low- and high-grade dysplasia characterisation.

	Low-grade dysplasia	High-grade dysplasia
Extension	–	Changes must involve more than two glands (except in tiny biopsies of polyps)
Low power magnification	Lack of architectural complexity suggests low-grade dysplasia throughout	Alterations have to be enough to be identified atlow power: complex architectural abnormalities, epithelium looks thick, blue, disorganised and “dirty”
Cytology/architecture	Does not combine cytological high-grade dysplasia with architectural high-grade features	Needs to combine high-grade cytological and high-grade architectural alterations
Architectural features*	Gland crowding, showing parallel disposition, with no complexity (no back-to-back or cribriforming); Global architecture may vary from tubular to villous	Complex glandular crowding and irregularity; Prominent budding; Cribriform appearance and back-to-back glands; Prominent intra-luminal papillary tufting
Cytological features**	Nucleus are enlarged and hyperchromatic, many times cigar-shaped; Nucleus maintain basal orientation (only up to the lower half of the height of the epithelium, although in some cases we can see glands with full-thickness nuclear stratification - this is not HGD if the archite- ture is bland); There is no loss of cell polarity or pleomorphism; No atypical mitosis; Maintained cytological maturation (mucin)	Noticeably enlarged nuclei, often with a dispersed chromatin pattern and evident nucleoli; Loss of cell polarity or nuclear stratification to the extent that the nuclei are distributed within all 1/3 of the height of the epithelium; Atypical mitoses; Prominent apoptosis/necrosis, giving the lesion a “dirty” appearance; Lack of cytological maturation (loss of mucin)

*Architectural features: gland morphology and placement; **Cytological features: cell level characteristics.

## Computational pathology

The digitisation of histopathology images opened many research opportunities for the field of computer-aided image analysis^23,24^. In fact, due to the high-resolution and complex nature of whole-slide image (WSI) evaluation, advances in image analysis are required, which provides the opportunity to apply and advance image processing techniques, as well as AI methodologies, such as machine learning (ML) and deep learning (DL) algorithms^3,4,23,24^. Moreover, the integration of AI into healthcare routines is a required milestone for the years to come, and thus, in terms of pathology-focused research, many DL architectures have been applied with many different tasks in mind, either to predict diagnoses or even to identify new biomarkers^3^.

Regarding the field of AI, and its application in computational pathology, DL models^25^, which consist of multiple layers of processing to learn different levels of data representation, are the most common and promising methods nowadays. The networks are composed of multiple layers, each with multiple nodes. The large numbers of hidden layers confer depth to the networks, hence the name. Each node performs a weighted sum of its inputs and then feeds it into a non-linear function, the result of which is passed forward as input to the following layer and so on until the last layer, which provides the network output. In this way, these models have the intrinsic ability to learn features, directly from the input data, useful for the task at hand^25^. In particular, convolutional neural networks (CNN) are applied to images and automatically extract features, which are then used to identify objects/regions of interest or to classify the underlying diagnosis^26^. In digital pathology, this type of models is used, for example, for mitosis detection^27,28^, tissue segmentation^29,30^, cancer grading^31,32^ or histological classification^33,34^.

### Challenges of whole-slide image analysis

Despite the popularity, clear potential, progress and good results of DL in computer vision, and in medical imaging in particular, researchers should carefully consider and manage its pros and cons^4,35^. Indeed, digital pathology brings some specific challenges that need to be addressed:

- *High dimensionality of data*. Histology images are extremely informative, but at the cost of high dimensionality, usually over $50,000\times 50,000$ pixels^35^. Hence, these images do not fit in the memory of a Graphics Processing Unit (GPU), which is usually needed to train DL models. Current methods either downsample the original image or extract multiple smaller patches, choosing between the cost of losing pixel information or losing spatial information, respectively;
- *Data variability*, due to the nearly infinite patterns resulting from the basic tissue types, and the lack of standardisation in tissue preparation, staining and scanning;
- *Lack of annotated data*, since extensive annotation is subjective, tedious, expensive and time-consuming;
- *Non-boolean diagnosis*, especially in difficult and rare cases, which makes the diagnosis process more complex;
- *Need for interpretability/explainability*, in order to be reliable, easily debugged, trusted and approved^4,35,36^.

Therefore, the research community has the opportunity to develop robust algorithms with high performance, transparent and as interpretable as possible, always designed and validated in partnership with pathologists. To this end, one can take advantage of some well-known techniques such as transfer learning (using pre-trained networks instead of training from scratch), weakly/unsupervised learning (analysing images only with slide-level labelling), generative frameworks (by learning to generate images, the algorithm can understand their main distinctive features) or multitask learning (learning interrelated concepts may produce better generalisations)^35^.

### Computational pathology on colorectal cancer

As mentioned earlier, the rise of DL and its application in computer vision has been critical to computer-aided diagnosis (CAD) research. Several researchers have sought to work alongside pathologists to improve or reduce the workload of diagnosing cancer using histopathological images. However, the development of AI applications for colorectal cancer (CRC) diagnosis on WSI is still limited, as noted by Thakur et al.^5^: of the 30 papers reviewed, only 20% have diagnosis as a final goal. In fact, the majority of the papers deal with a wide variety of tasks, with a particular focus on tissue segmentation, the goal of 62% of the reviewed papers^5^. Last year, Wang et al.^6^ also published a review on the application of AI to CRC diagnosis and therapy, reflecting the same trend. However, CRC diagnosis is a growing application, with an increasing number of publications in recent years. In the next section, we collect and describe the published works on CRC diagnosis, with a particular focus on slide diagnosis (Table 2), but also summarising some works using partial regions of tissue (region crops or tiles) without aggregation for WSI.

#### CRC diagnosis on WSI

In 2012, Kalkan et al.^37^ proposed a method for CRC automatic detection from Haematoxylin and Eosin (H&E) slides, combining textural and structural features of smaller patches ($1024\times 1024$ pixels). Firstly, the patches are classified into normal, inflamed, adenomatous or cancer with a k-NN classifier, based on local shape and textural features, such as Haralick features, Gabor filters features and colour histograms features. Then, the (up to) 300 patches representing the slide are summarised in the average probabilities for all the four primary classes, and used as a feature vector for a logistic-linear regressor, to obtain a final slide diagnosis: normal or cancer. The proposed method was trained on 120 H&E stained slides and achieved an Area Under the Curve (AUC) of 0.90 and an average accuracy of 87.69%, with accuracies of 79.17% and 92.68% for cancer and normal slides, respectively. Similarly, using traditional computer vision techniques, Yoshida et al.^38^ presented an approach to classify CRC H&E slides into 4 types: non-neoplastic, adenoma, carcinoma and unclassifiable. For each WSI, all tissue regions are identified, summing 1328 sections from 1068 H&E slides. Then, each section is processed for blur detection and colour normalisation before the analysis in two steps: cytological atypia analysis and structural atypia analysis. In the first step, the method proposed by Cosatto et al.^39^ is used, based on multiple instance learning (MIL) formulation using a Multilayer Perceptron (MLP), to grade the degree of cytological alteration of the tissue (high or low).

Then, the image is classified into low atypia, intermediate atypia, high atypia or unclassifiable, based on structural nuclear features and cytoplasmatic features, extracted from consecutive ROIs, that are summarised by the mean-square of the top 3 ROIs. Finally, each image is classified based on the combination of structural atypia analysis result (high, intermediate or low) and the cytological atypia analysis result (high or low), given that carcinoma presents higher atypia values. The model has an undetected carcinoma rate of 9.3%, an undetected adenoma rate of 0.0% and an over detection proportion of 27.1%.

The first DL application model was presented in 2017, by Korbar et al.^40^, to automatically classify colorectal polyps on H&E stained slides into five classes: normal, hyperplastic, sessile serrated, traditional serrated adenoma, tubular adenoma and tubulovillous/villous adenoma. The 697 H&E stained slides (annotated by a pathologist) were cropped into ROIs of $811\times 984$ pixels (mean size), and then divided into overlapping smaller patches. These patches were classified using the ResNet-152 and the prediction of the slide was obtained as the most common colorectal polyp class among all patches of the slide. However, if no more than five patches are identified with the most common class, with a confidence higher than 70%, the slide is classified as normal. The proposed system achieved 93.0% accuracy, 89.7% precision, 88.3% recall and 8.8% F1-score. Later, the authors proposed a visualisation method^41^, based on this approach, to identify highly-specific ROIs for each type of colorectal polyps within a slide, using the Guided Grad-CAM method^42^ and a subset of data (176 H&E colorectal slides).

In 2020, several authors presented solutions for CRC diagnosis, with varying degrees of detail. Iizuka et al.^43^ proposed the combination of a Inception-v3 network with a recurrent neural network (RNN) to classify H&E colorectal WSI into non-neoplastic, adenoma and adenocarcinoma. Each slide was divided into patches of $512\times 512$ pixels (at 20X magnification, with a sliding window of 256 pixels) and assigned to one of the three diagnostic classes. Then, all tiles are aggregated using a RNN, trained to combine the features outputted by the CNN. The dataset consists of subsets from two different institutions, summing 4536 WSIs. Moreover, the model was also evaluated on a subset of 547 colon surgical resection cases from The Cancer Genome Atlas (TCGA) repository^44^, containing adenocarcinoma and normal samples (TCGA-COAD collection). On the private dataset, the proposed approach measured AUCs of 0.962 and 0.992 for colorectal adenocarcinomas and adenomas, respectively. On the public dataset, the model achieved an 0.982 AUC for adenocarcinomas. It is noteworthy that the authors report that, since the samples from the external subset are much larger than the biopsies used for training, the RNN aggregation was replaced by a max-pooling aggregation. Meanwhile, Wei et al.^45^ aimed to identify five types of polyps in H&E stained colorectal slides: normal, tubular adenoma, tubulovillous or villous adenoma, hyperplastic polyp, and sessile serrated adenoma. To train the model, the authors used 509 slides (with annotations of relevant areas by five specialised pathologists) and for further testing, they used an external set of 238 slides, obtained from different institutions. The model consists of an ensemble of the five versions of ResNet (namely, networks with 34, 50, 101, and 152 layers) to classify tiles of $224\times 224$ pixels (at 40X magnification). Then, the patches are combined with a hierarchical classifier to predict a slide diagnosis. Based on the predicted tile classes, the model first classifies a polyp as adenomatous or serrated, by comparing the frequency of tiles classes (tubular, tubulovillous, or villous vs. hyperplastic or sessile serrated). Adenomatous polyps with more than 30% tubulovillous or villous adenoma tiles are classified within this class and the remaining are classified as tubular adenoma. Serrated polyps with more than 1.5% of sessile serrated tiles are classified within this class and the remaining are classified as hyperplastic. The thresholds were set with a grid search over the training set, reaching an accuracy of 93.5%, on the internal test set, and 87.0% on the external test set.

Moreover, also during last year, two other authors proposed segmenting colorectal tissue simultaneously with the diagnosis. Song et al.^46^ presented an approach based on a modified DeepLab-v2 network on $640\times 640$ pixel tiles, at a 10X magnification. The dataset consists of 411 annotated slides, labelled as colorectal adenomas or normal mucosa (which includes chronic inflammation), and a subset of 168 slides collected from two other institutions, to serve as an external test. The authors modified the DeepLab-v2 network by introducing a skip layer that combines the upsampled lower layers with the higher layers, in order to retain semantic details of the tiles. Then, the 15th largest pixel-level probability is used for the slide-level prediction. In the inference phase, the slide is decomposed into tiles of 22002200 pixels. The proposed approach achieved an AUC of 0.92 and, when tested on the independent dataset, an accuracy over 90%. In turn, the model of Xu et al.^47^ was trained on a set of 307 slides (normal and CRC), with tissue boundaries manually annotated by a pathologist, achieving a mean accuracy of 99.9% for normal slides and 93.6% for cancer slides, and a mean dice coefficient of 88.5%. For further testing, the model was also evaluated on an external set of 50 CRC slides and achieved a mean accuracy of 87.8% and a mean Dice coefficient of 87.2%. The method uses the Inception-v3 architecture, pre-trained on the ImageNet dataset, to classify patches of $768\times 768$ pixels, resized to $299\times 299$ pixels. The final tumour regions and slide diagnosis are obtained by thresholding the tile predictions: tiles with tumour probability above 0.65 are considered as cancer.

**Table 2:** Literature overview on colorectal whole slide image diagnosis.

Author	Year	Task	Dataset	Description	Results
Kalkan et al.^37^	2012	CRC detection (normal vs cancer)	120 H&E slides (tile annotations)	1024 × 1024 px tiles; k-NN classifier + Logistic-linear classifier	Acc.: 87.69%; AUC: 0.90
Korbar et al.^40^	2017	Polyp classification (6-class): normal, hyperplastic, sessile serrated, traditional serrated, tubular and tubulovillous/villous	697 H&E slides (annotated)	811 × 984 px ROIs (mean size); ResNet-152 + argmax of tile class frequency	Acc.: 93%; Precision: 89.7%; Recall: 88.3%; F1-score: 88.8%
Yoshida et al.^38^	2017	CRC classification (4-class): unclassifiable, non-neoplastic, adenoma and CA	1068 H&E slides (w/ labelled tissue sections)	Tissue sections crop + cytological atypia analysis + structural atypia analysis + overall classification	FNR (CA): 9.3%; FNR (adenoma): 0%; FPR: 27.1%
Iizuka et al.^43^	2020	CRC classification (3-class): non-neoplastic, AD and ADC	4536 H&E slides (annotated) + 547 H&E slides from TCGA-COAD collection	512 × 512 px tiles at 20×; Inception-v3 + RNN	AUC: 0.962 (ADC), 0.993 (AD); AUC (TCGA-COAD subset): 0.982 (ADC)
Song et al.^46^	2020	Colorectal adenoma detection (normal vs adenoma)	411 H&E slides (annotated) + external set: 168 H&E slides	640 × 640 px tiles at 10×; Modified DeepLab-v2 + 15th largest pixel probability	AUC: 0.92; Acc. (external set): >90%
Wei et al.^45^	2020	Polyp classification (5-class): Normal, hyperplastic, tubular, tubulovillous/villous, sessile serrated	508 H&E slides (annotated) + external set: 238 H&E slides	224 × 224 px tiles at 40×; ResNet models ensemble + hierarchical classification	Acc.: 93.5%; Acc. (external set): 87%
Xu et al.^47^	2020	CRC detection (normal vs cancer)	307 H&E slides (annotated) + 50 H&E slides (external set)	768 × 768 px tiles; Inception-v3 + tiles tumour probability thresholding	Acc.: >93%; Acc. (external set): >87%

*CRC*: Colorectal Cancer; *AD*: Adenoma; *CA*: Carcinoma; *ADC*: Adenocarcinoma; *H&E*: Haemotoxylin & Eosin; *px*: pixels; *k-NN*: k Nearest Neighbours; *ROI*: Region of Interest *CNN*: Convolutional Neu- ral Network; *SVM*: Support Vector Machine; *MLP*: Multi-Layer Perceptron; *MIL*: Multiple Instance Learning; *Acc.*: Accuracy; *AUC*: Area Under the ROC Curve; *FNR/FPR*: False Negative/Positive Rate

While some of the reported results are impressive and show high potential, there are still some obvious shortcomings that need to be addressed. One of the issues is model evaluation: most of the papers analysed have not used any form of external evaluation on public benchmark datasets, as can be seen by the dataset descriptions on Table 2. This validation is necessary to understand and compare the performance of models that, otherwise, cannot be directly compared to each other due to the use of distinct datasets. It also limits the study of robustness of the model when it is exposed to data from sources other than those used for training. On the other hand, as with any DL problem, the size of the dataset is crucial. Although, as mentioned earlier, it is expensive to collect the necessary amount of data to develop a robust model, it is noticeable that the reviewed articles could greatly benefit from an increase in the volume of data, since most of the works are trained on only a few hundred slides (Table 2). Describing and sharing how the data collection and annotation processes were performed is also crucial to assess the quality of the dataset and the quality of the annotations. For example, the number of annotators, their experience in the field, and how their discrepancies were resolved. However, this description was not a common practice in the articles reviewed. Moreover, comparing models becomes more complicated when one realises that the number of classes used for the classification tasks is not standardised across published work. Therefore, together with the difference in the kind of metrics presented, direct comparisons should be made with caution.

#### Other CRC classification approaches

Despite the small number of published works on colorectal WSI diagnosis (Table 2), there is a myriad of other articles also working on CRC classification using information from smaller tissue regions, that can be exploited as a basis for general diagnostic systems. Despite the different task, these works that use image crops, or even small patches^48–52^, can be leveraged for slide diagnosis, in combination with aggregation methods that combine all the extracted information in a single prediction.

As for WSI classification, there are also approaches for crop images classification based on traditional computer vision methods or DL models, and even a combination of both. In 2017, Xu et al.^53^ proposed the combination of an Alexnet (pre-trained on ImageNet dataset) as feature extractor and a SVM classifier to develop both a binary (normal vs. cancer) and a multiclass (CRC type) classification approach for cropped images (variable size, 40X magnification) from CRC H&E slides. The latter goal is to distinguish between 6 classes: normal, adenocarcinoma, mucinous carcinoma, serrated carcinoma, papillary carcinoma cribriform adenocarcinoma. Each image is divided into overlapping patches of $672\times 672$ pixels (then resized to 224224 pixels), from which 4096-dimensional feature vectors are extracted. For cancer detection, features are selected based on the differences between positive and negative labels: the top 100 feature components (ranked from the largest differences to the smallest) are kept. Then the final prediction is obtained with a linear SVM (one-vs-rest classification for CRC type). The CRC detection model has an accuracy of 98% and the CRC type classification model has an accuracy of 87.2%, trained on 717 image crops. Already in 2019, Yang et al.^54^ and Ribeiro et al.^55^ proposed works based on color and geometric features, and classical ML methods, to classify CRC. With colour pictures ($350\times 350$ pixels, 20× magnification) from H&E stained colorectal tissue sections (labelled and marked by professional pathologists), Yang et al.^54^ proposed a method based on sub-patch weight color histogram features, the RelicfF based forward selection algorithm and a Morlet wavelet kernel-based least squares SVM classifier. The method was developed using a total of 180 images and obtained an AUC and accuracy of 0.85 and 83.13%, respectively. Ribeiro et al.^55^ associated multidimensional fractal geometry, curvelet transforms and Haralick features and tested several classifiers on 151 cropped images ($775\times 522$ pixels, 20X magnification) from 16 H&E adenocarcinoma samples. The best result, an AUC of 0.994, was achieved with multiscale and multidimensional percolation features (from curvelet sub-images with scale 1 and 4), quantifications performed with multiscale and multidimensional lacunarity (from input images and their curvelet sub-images with scale 1) and a polynomial classifier.

Regarding DL models, there are also several proposed approaches for several CRC classification tasks. In 2017, Haj-Hassan et al.^56^ proposed a method based on multispectral images and a custom CNN to predict 3 CRC types: benign hyperplasia, intrapithelial neoplasia and carcinoma. From the H&E stained tissue samples of 30 patients, 16 multispectral images of $512\times 512$ pixels are acquired, in a wavelength range of 500–600 nm. After a CRC tissue segmentation with an Active Contour algorithm, images are cropped in smaller tiles of $60\times 60$ pixels (with the same label of the slide) and fed to a custom CNN (input size of $60\times 60\times 16$), reaching an accuracy of 99.17%. In 2018, Ponzio et al.^57^ adapted a pre-trained VGG16 net for CRC classification into adenocarcinoma, tubuvillous adenoma and healthy tissue. They used tissue subtype large ROIs, identified by a skilled pathologist from 27 H&E stained slides of colorectal tissue from a public repository^58^, that were then cropped into $1089\times 1089$ patches, at a magnification level of 40×. By freezing the weights up to the most discriminative pooling layer (determined by t-SNE) and training only the final layers of the network, the solution provided a classification accuracy over 90%. The system was evaluated at two levels: the patch score (fraction of patches that were correctly classified) and patient score (per-patient patch score, averaged over all cases), that reached 96.82% and 96.78%, respectively. In 2019, Sena et al.^59^ proposed a custom CNN to classify four stages of CRC tissue development: normal mucosa, early pre-neoplastic lesion, adenoma and carcinoma. The dataset consist of 393 images from H&E colorectal slides (20× magnification), cropped into nine subimages of $864\times 548$ pixels. For further validation on significantly different images, the authors also used the GLaS challenge dataset^60,61^, with 151 cropped images. Since both datasets differ on resolution, the GLaS images were resized with bi-cubic interpolation and centrally cropped. The proposed method obtained an overall accuracy of 95.3% and the external validation returned an accuracy of 81.7%. Meanwhile, Zhou et al.^62^ proposed a pipeline to classify colorectal adenocarcinomas, based on the recent graph neural networks, converting each histopathological image into a graph, with nucleus and cellular interactions being represented by nodes and edges, respectively. The authors also propose a new graph convolution module, called Adaptive GraphSage, to combine multilevel features. With 139 images ($4548\times 7520$ pixels, 20x magnification), cropped from WSI labelled as normal, low grade and high grade, the method achieved an accuracy of 97%. For the same classification task, in 2020, Shaban et al.^63^ proposed a context-aware convolution neural network to incorporate contextual information in the training phase. Firstly, tissue regions ($1792\times 1792$ pixels) are decomposed in local representations by a CNN ($224\times 224$ pixels input), and the final prediction is obtained by combining all contextual information with a representation aggregation network, considering the spatial organisation of smaller tiles. This method was developed on 439 images ($\sim$ $5000\times 7300$ pixels, 20× magnification) and achieved an average accuracy of 99.28% and 95.70% for a binary and three class setup, respectively.

## Feasibility study on the use of weakly annotated and larger datasets for CRC diagnosis

Collecting and labelling data for computational pathology problems is a lengthy and expensive process. As seen in the previous section, research is often conducted on small datasets containing a high granularity of annotations per sample. Despite the benefits of detailed annotations, researchers have recently turned their attention to weakly-supervised approaches. These approaches, notwithstanding the simplified annotation, can leverage larger datasets for learning. More importantly, weakly-supervised learning techniques are less prone to bias in data collection. Performance is also evaluated on a more extensive test set, and thus, the behaviour of the model in the real-world can be generalised much more accurately. In this section, we conduct a feasibility study on the use of efficiently annotated datasets to drive the development of computer-aided diagnosis (CAD) systems for colorectal cancer (CRC) from whole-slide images (WSI). We attempt to answer the question of the required dimension of the dataset, as well as the extension of annotations, to enable the robust learning of predictive models. We also analyse the advantage of using a loss function adapted to the ordinal nature of the classes corresponding to the CRC scores.

### Datasets

This feasibility study was conducted on two datasets: the first contains colorectal haematoxylin & eosin (H&E) stained slides (CRC dataset), whereas the second includes prostate cancer H&E-stained biopsy slides (PCa dataset). As mentioned in “Computational pathology on colorectal cancer” section, there is a shortage of large public datasets containing CRC WSIs and most of the existing ones are based on cropped regions instead of entire slides. Hence, we relied on a PCa dataset that, while not fully transferring to colorectal use case, is one of the largest WSI datasets publicly available. This amount of data allowed us to study the data requirements of a weakly supervised approach and how the performance evolved with the growth of the dataset.

#### CRC dataset

The CRC dataset contains 1133 colorectal biopsy and polypectomy slides and is the result of our ongoing efforts to contribute to CRC diagnosis with a reference dataset. We aim to detect high-grade lesions with high sensitivity. High-grade lesions encompass conventional adenomas with high-grade dysplasia (including intra-mucosal carcinomas) and invasive adenocarcinomas. In addition, we also intend to identify low-grade lesions (corresponding to conventional adenomas with low-grade dysplasia). Accordingly, we created three diagnostic categories for the algorithm, labelled as non-neoplastic, low-grade and high-grade lesions (Table 3). In the non-neoplastic category, cases with suspicion/known history of inflammatory bowel disease/infection/etc. were omitted. We selected conventional adenomas as they were the largest group on daily routine (serrated lesions, and other polyp types, were omitted).

**Table 3:** CRC dataset class definition.

Algorithm data classes	Pathological diagnosis
Non-neoplastic	Normal CR mucosa, non-specific inflammation, hyperplasia
Low-grade lesion	Low-grade conventional adenoma
High-grade lesion	High-grade conventional adenoma and invasive adenocarcinoma

**Figure 3:**
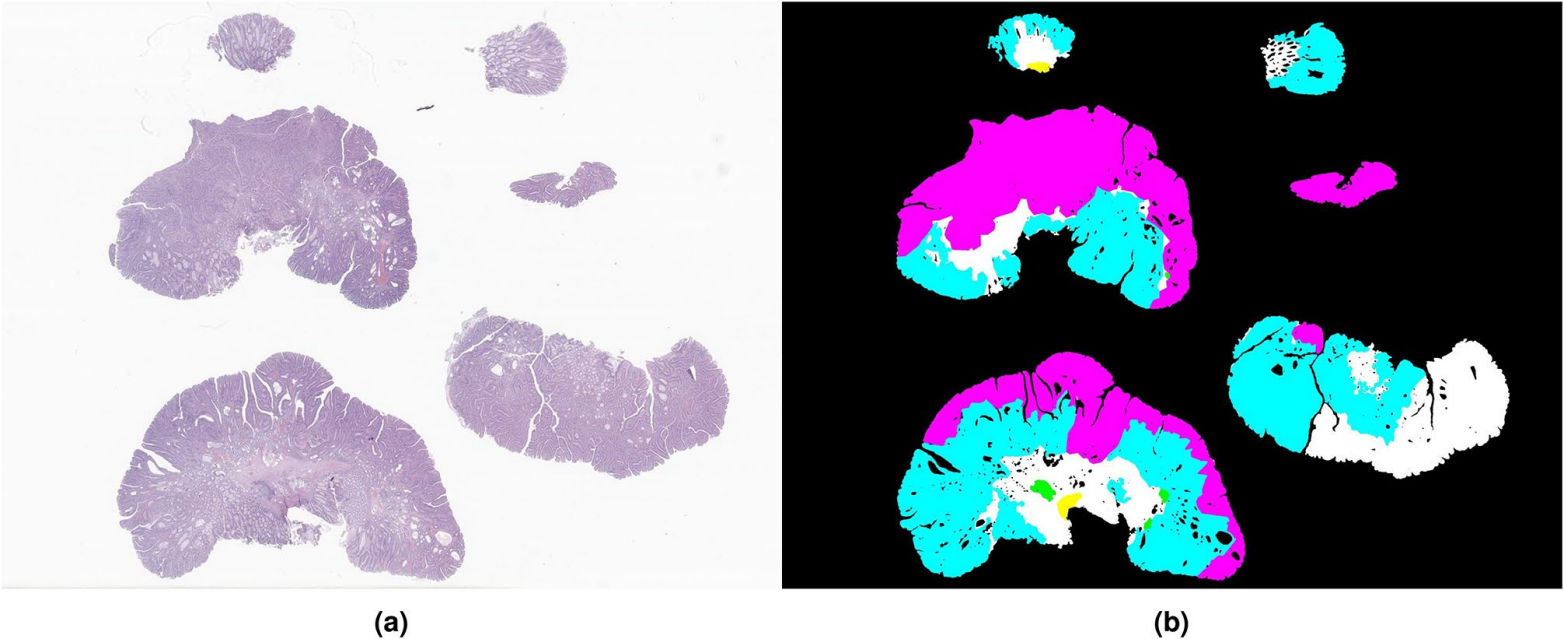
Example of a whole-slide (**a**) from the CRC dataset. Manual segmentations (**b**) include regions annotated as non-neoplastic (white), low-grade lesions (blue), high-grade lesions (pink), linfocytes (green) and fulguration (yellow).

All cases were retrieved from the data archive of IMP Diagnostics laboratory, Portugal, and were digitised by 2 Leica GT450 WSI scanners, and evaluated by one of two pathologists (Fig. 3a). Data collection and usage was performed in accordance with national legal and ethical standards applicable to this type of data. Since the study is retrospectively designed, no protected health information was used and patient informed consent is exempted from being requested.

Diagnostics were made using a medical grade monitor LG 27HJ712C-W and Aperio eSlide Manager software. When reviewing the cases, most diagnosis were coincident with the initial pathology report and no further assessment was made. In case of difference, the case was rechecked and decided between the two pathologists. A small number of cases (n=100) were further annotated with region marks (Fig. 3b) by one of the pathologists and then rechecked by the other, using the Sedeen Viewer software^64^.

Corrections were made when considered necessary by both. For complex cases, or when agreement could not be reached, both the label and/or annotation were reevaluated by a third pathologist. Case classification followed the criteria previously described in “Dysplasia grading” section. Accordingly, cases with only minimal high-grade dysplasia (HGD) areas (only one or two glands), or with areas of florid high-grade cytological features but without associated worrisome architecture, were kept in the low-grade dysplasia class, as well as cases with cytological high-grade dysplasia only seen on the surface. It is worth noting that some cases may be more difficult to grade and have to be decided on a case-by-case basis, preferentially by consensus. Additionally, as recommended by the World Health Organization (WHO), intramucosal carcinomas were included in the HGD cases^20,65^.

**Figure 4:**
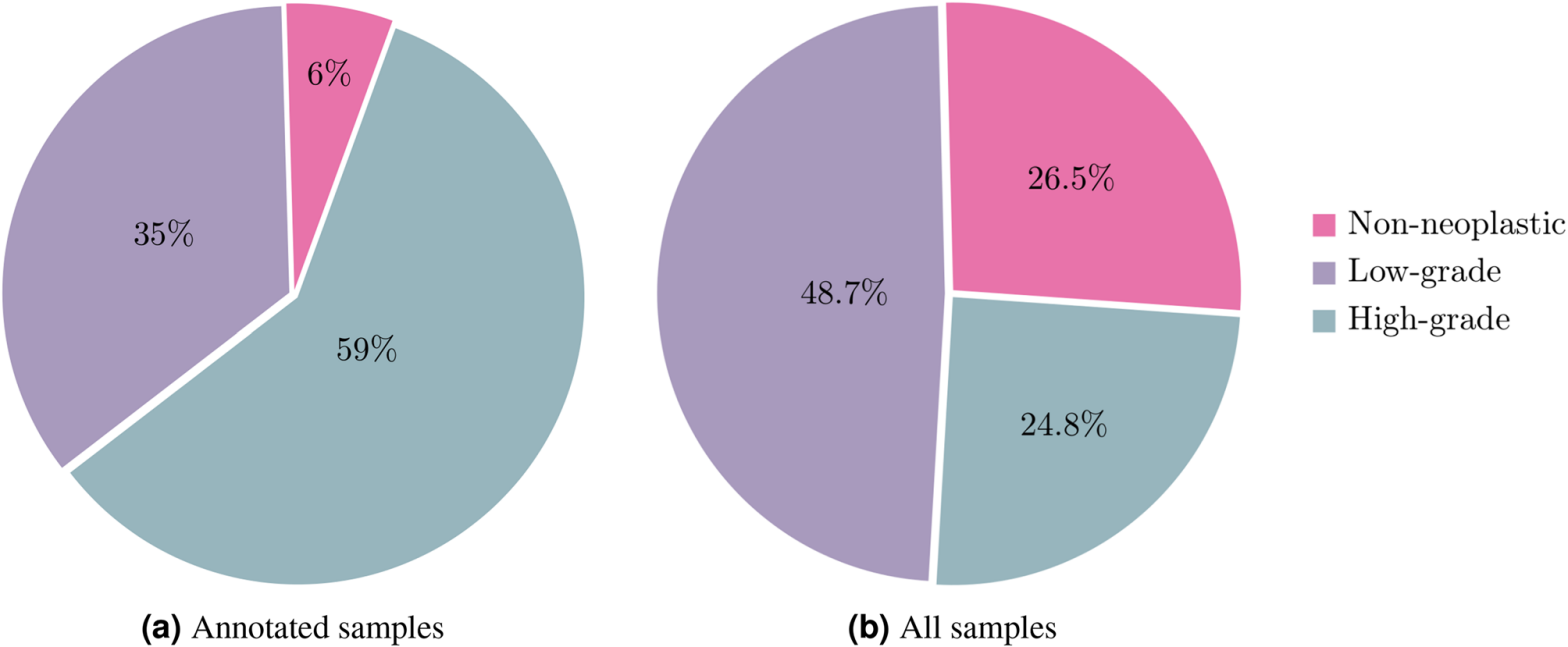
Slide classes distribution on CRC dataset.

Regarding the distribution of slide labels, while the annotated samples are considerably imbalanced, as seen in Fig. 4a, when combined with the non-annotated samples, the distribution of the labels is significantly more even. Figure 4b shows this final distribution and it is closer to what is seen in clinical practice. Moreover, it was important to fully annotate cases that are especially difficult or high-grade, so the model can learn more about these critical cases. The CRC dataset was used not only to develop the proposed methodology, but also to evaluate the relevance of annotations in a model pre-training: can a small set of annotated images leverage the overall performance of the weakly supervised model?

#### PCa dataset

Besides the influence of the level of annotations, we also aimed to evaluate the proposed classification methodology on a larger dataset, also with a multiclass formulation, to investigate the impact of the dataset size on the performance of the algorithm. In this sense, we used 9,825 PCa biopsy slides from the dataset of the Prostate cANcer graDe Assessment (PANDA) challenge^66^. The available full training set consists of 10,616 WSI of digitized H&E stained PCa biopsies (we excluded cases with some type of error) obtained from two centres: the Radboud University Medical Centre and the Karolinska Institute, and includes both labelling and tissue segmentation. Each image is labelled with the correspondent ISUP grade and includes tissue annotation, differentiating tumour areas from normal tissue. The International Society of Urological Pathology (ISUP) grading system is the currently score to grade PCa, which is based on the modified Gleason system (a score based on glandular architecture within the tumour), providing accurate stratification of PCa^67^.

The PCa dataset contains six different labels, corresponding to the five ISUP grades and the normal label, whereas the CRC dataset has only three different labels. Histopathological slides are quite different for different types of cancer, for instance, the quantity of tissue varies significantly. The images require some pre-processing that creates the tiles from the WSI. Such processing removes the background, and thus, tissue variations deeply affect the number of tiles of one slide. Table 4 displays an illustrative example of this, by comparing the number of tiles and the mean number of tiles per slide included in both datasets. The average number of tiles per slide is approximately 82x and 45x higher, respectively on the CRC annotated subset and on the complete dataset, when compared to the PCa dataset. Because of this variation in tissue proportion, despite having 8.6x more slides, the PCa dataset still has around 5x fewer tiles.

**Table 4:** Comparison between the number of tiles extracted from the PCa slides and the CRC slides.

Dataset	# Slides	# Tiles	Mean # Tiles per slide
PCa	9825	253,291	25.78
CRC all	1133	1,322,596	1167.34
CRC annotated	100	211,235	2112.35

### Methodology

**Figure 5:**
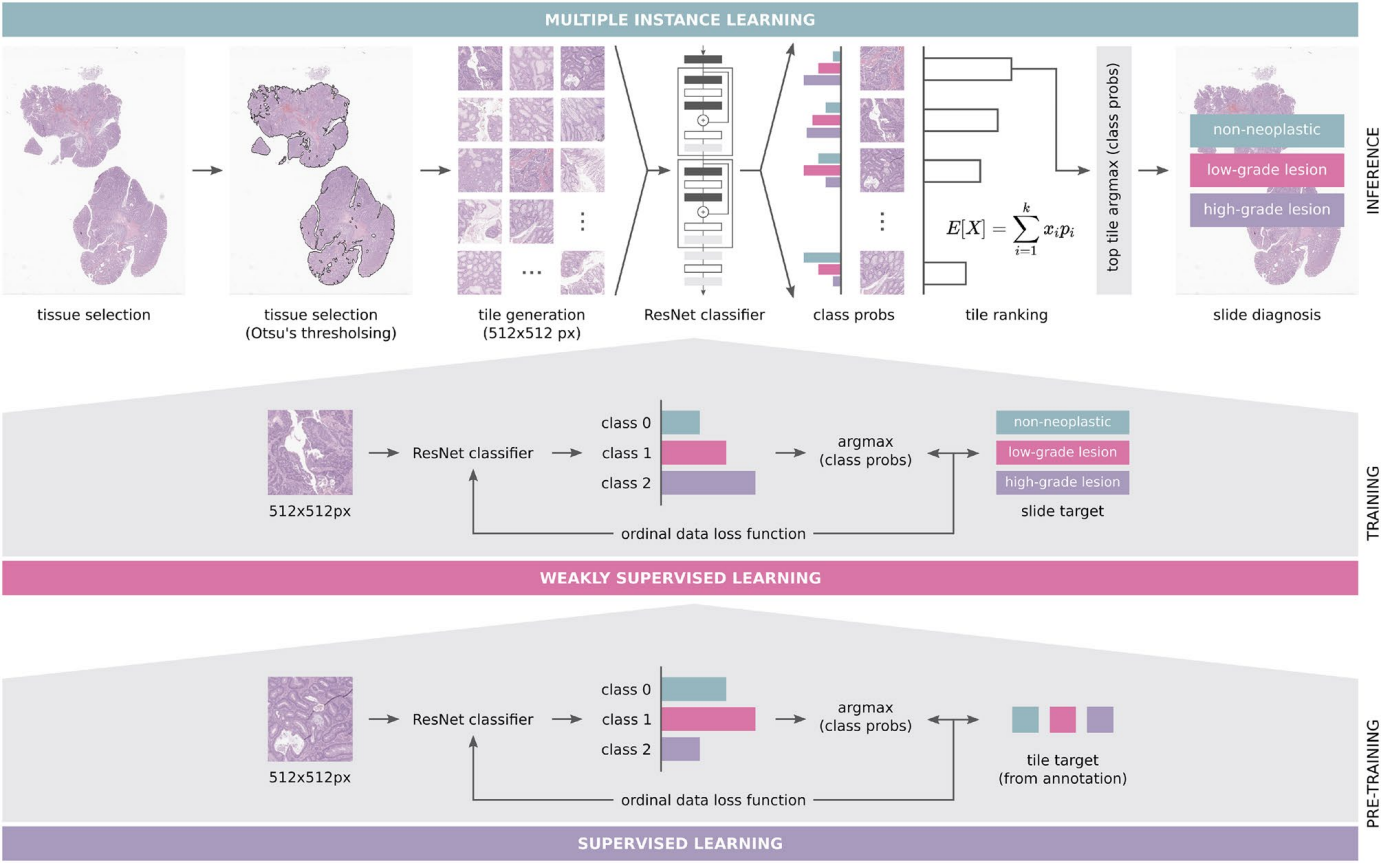
Proposed workflow for colorectal cancer diagnosis on whole-slide images.

Traditional supervised learning techniques would require all the patches extracted from the original image to be labelled. However, cancer grading (in clinical practice) aims to classify the whole slide image, not individual tiles. Moreover, labelling the tiles represents a significant effort with regards to the workload of the pathologists. Therefore, techniques such as multiple instance learning (MIL), have been adapted to computational pathology problems^68–70^. MIL only requires slide level labels and the original supervised problem is converted to a weakly-supervised problem. The nature of the problem allows the implementation of this technique knowing that, if a WSI is classified with a label Y, no tile belongs to a more severe class than Y and at least one tile belongs to the label Y. Therefore, using the MIL concept, we propose a workflow (Fig. 5) based on the work of Campanella et al.^69^ with several adaptations:

1. *Ordinal labels:* First, the problem at hand has a multiclass formulation, whereas the original had only two labels. In order to contextualise the premises of the MIL method and the clinical information, the labels must not be seen as independent and their relation must be modelled. For instance, normal tissue is closer to low-grade lesions than to high-grade dysplasias. Thus, there is an order regarding labels.
2. *Removal of recurrent aggregation:* The original approach leveraged a Recurrent Neural Network (RNN) to aggregate predictions of individual tiles into a final prediction. All the tests conducted for the feasibility results did not show any benefit of having this RNN aggregation, in fact, the performance degraded. Thus, it was removed from the pipeline.
3. *Tile ranking using the expected value:* Using a single tile for the prediction requires a ranking rule in order to select the most representative of potentially thousands of tiles. Since the problem is non-binary, the original rule is not applicable^69^. Therefore, to create a ranking of tiles that are meaningful for the final prediction, the backbone network is used to compute the outputs of each tile and the expected value is then computed from these outputs:
  $$\begin{aligned} tile\_score = \sum _{i=1}^{n\_classes}{x_{i}\times p_{i}} \end{aligned}$$
  with n_classes the number of classes, $x_i$ the class, $p_i$ the correspondent probability;
4. *Loss function:* The problem includes ordinal labels, so the minimisation of the cross-entropy fails to fully capture the model’s behaviour. As mentioned before, the distance between labels is different and cross-entropy treats them as if they are equally distant. Thus, in an attempt to increase the performance of the initial baseline experiments the model is now optimised to minimise an approximation to the Quadratic Weighted Kappa (QWK)^71^.

#### Training details

The setup of the experiments was similar across datasets: ResNet-34 as the backbone, batch-size of 32, the Adaptive Moment Estimation (Adam) algorithm with a learning rate of $1\times 10^{-4}$ as optimiser, tiles of $512\times 512$ pixels that include 100% of tissue and mixed-precision from the Pytorch available package. Only one tile was used for predicting the label of the slide (MIL formulation), thus the training set was only regarding the selected tile. As for hardware, all the experiments were conducted using an Nvidia Tesla V100 (32 GB) GPU.

### Experimental results and discussion

In deep learning problems, it is not always trivial to determine the required dataset size to achieve the expected performance. Usually, it is expected that increasing the size of the dataset increases the model performance. However, this is not always true. Hence, to fully understand the impact of the dataset size in the computational pathology domain, the developed approach was trained on several subsets of the original PCa training set with different sizes: 80, 160, 500, 1000, 2500, 5000 and 8348 (complete training set). For a fair comparison, all the experiments were evaluated on the same test set, that included 1477 slides (15% of the total dataset) independent from the training set. As can be seen in Table 5, the model is able to leverage more data in order to achieve better performance. Moreover, in line with these results, Campanella et al.^69^ stated that for the MIL approach to work properly, the dataset must contain at least 10,000 samples. In our experiments, the performance with 5000 slides was already close to the best performance.

To further infer the generalisation capability, an extra model was trained on 80 slides and evaluated on 20 slides randomly sampled from the 1477 test set. As seen in Table 6, as expected, when the size of the test set increases, the performance rapidly degrades, nursing the concerns and requirements for larger datasets. It is also worth noting that the performance of the model is considered poor in terms of accuracy scores. The QWK, on the other hand, records reasonable values. This difference in performance means that, while the model misclassifies about 40% of the slides, it classifies them with neighbour classes of the ground-truth. One possible reason for this could be the noise present in the labels of this specific dataset.

**Table 5:** Evolution of the model performance when trained on subsets of the PCa dataset with different sizes, keeping the test set size constant (n=1,477).

# Train slides	# Train tiles	QWK score	Accuracy (%)
80	1919	0.497	32.36
160	3851	0.586	37.71
500	12,714	0.628	41.28
1000	25,757	0.692	47.66
2500	64,697	0.738	50.03
5000	129,734	0.771	58.43
8348	215,116	**0.789**	**59.40**

Bold values indicate best results

**Table 6:** Performance comparison of the model trained on a subset of the PCa dataset, when evaluated on test sets with different sizes.

Dataset	# Train slides	# Test slides	# Train tiles	# Test tiles	QWK score
PCa	80	1,477	1,919	38,175	0.497
PCa	80	20	1,919	579	**0.591**

Bold value indicates best results

The third set of experiments explores the potential to leverage the annotations of a subset of data in order to improve the performance of the overall MIL method. Table 7 shows the results of the best epoch of each of the experiments. There are notable performance gains in both the accuracy and the QWK score as the number of training samples increases. However, perhaps the most exciting performance gain is related to the pre-training of the backbone network on the 100 annotated samples for only two epochs before the start of the MIL training. This experiment is able to outperform the best epoch of the experiment without pre-training in only 7 epochs, in other words, 12 hours of training, with 84.94% accuracy and 0.803 QWK score. Moreover, these values kept increasing until the last training epoch, reaching an accuracy and QWK score of 88.42% and 0.863, respectively. The final results presented in Table 7 can be extended with a sensitivity to lesions of 93.33% and 95.74% for the last two entries respectively. The training set comprises 874 samples (100 annotated and 774 non annotated), whereas the test set has 259 WSI.

**Table 7:** Performance of the model on the different experiments on the CRC dataset.

Dataset	Pre-train	QWK score	Accuracy (%)	Convergence Time (Epoch)
CRC annotated (n=100)	No	0.583	75.00	6.5 h (13)
CRC All (n=1,133)	No	0.795	84.17	2 days and 19 h (27)
CRC All (n=1,133)	Yes	**0.863**	**88.42**	4 days (40)

Bold values indicate best results

**Figure 6:**
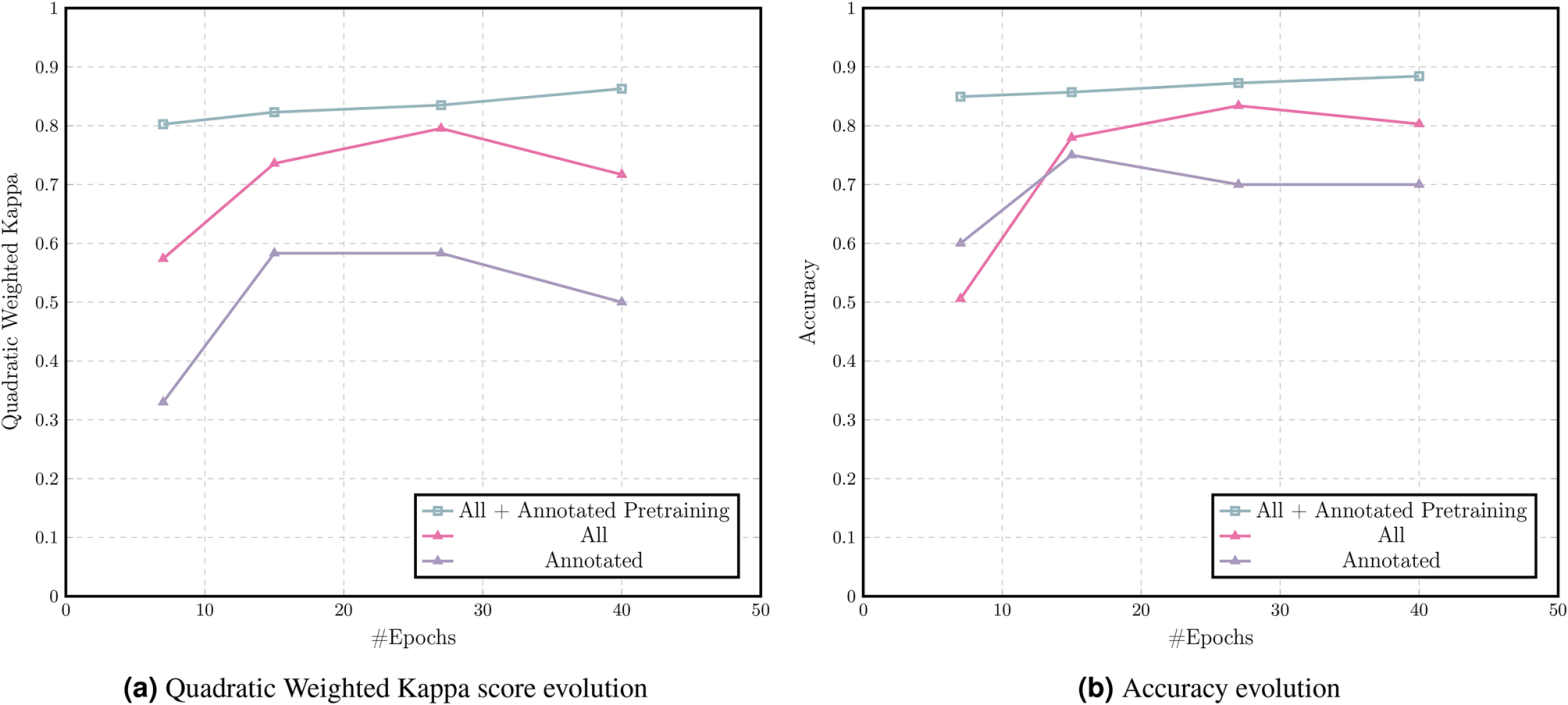
Performance evaluated on CRC dataset.

The results shown in Fig. 6a,b, respectively for the QWK and the accuracy, are representative of the gains that both the number of samples and the use of annotations bring to the model. Moreover, the use of annotations appears not only to speed up convergence at high values, but also to increase the model’s ability to learn at further epochs.

The finding in these feasibility results supports the need for larger datasets. Not only that, but it also increases the confidence in the performance of weakly-supervised learning techniques, especially if it is possible to include at some point some supervised training to propel the performance even more. It is expected that these novel techniques and larger datasets converge to models that are closer to be deployed for clinical practice.

## Conclusions

Despite the growing popularity and availability of computational pathology works, there are relatively few published works on colorectal cancer (CRC) diagnosis from whole-slide images (WSI), as shown in “CRC diagnosis on WSI” section. Moreover, the reported results are based on relatively small and private datasets, which renders them fragile and makes direct comparisons not so fair. The reviewed papers on CRC diagnosis leverage different techniques and approaches to solve the same problem, from attention to graph neural networks and even weakly-supervised learning. However, there is still room for progress in each of these techniques, both individually and in combination. Nevertheless, it is difficult to perform a proper evaluation when the size of the dataset does not favor the use of these techniques and limits the research. Therefore, the first and perhaps most crucial step for the further development of computer-aided diagnosis (CAD) systems for CRC is to establish a large and meaningful dataset. As studied in “Feasibility study on the use of weakly annotated and larger datasets for CRC diagnosis” section, increasing the number of WSI in the training data leads to an increase in performance, as does detailed annotation of, at least, part of the dataset.

Nonetheless, the construction of larger datasets with extensive annotations is not an easy and expeditious task. Hence, there is still a plethora of techniques to be explored with weakly labelled datasets. One of these tasks is known as multiple instance learning (MIL) and while it has been employed several times on these type of problems, it can still be improved to achieve more accurate results. As shown in “Experimental results & Discussion” section, the performance of MIL systems is greatly improved with a pre-training on the 10% of the dataset that is annotated. Future research is needed to delineate the lowest percentage of the dataset that needs to be annotated so this approach improves both accuracy and convergence times. Moreover, there are other techniques that can leverage weak labels. For instance, self-supervised learning methods leverage all the pixels of the images as “labels”. On this note, it is important to also evaluate if there is a feasible way to combine the benefits of both methodologies.

Since the main goal of deep learning (DL) in computational pathology is to develop a solution that can be deployed in a clinical environment, it is important to develop it in a similar fashion to the clinical practice, in other words, to handle the same type of data given to pathologists, WSI. In that sense, the work proposed in this paper is considerably more in line with the end goal of CAD systems for computational pathology: our proposal can be directly applied to a lab workflow.

### Future work

Other approaches to be explored in the future are based on improving the supervised pre-training stage. For example, could it be possible to use pre-training, not as a separate initial process, but, instead, use annotated data as an extra-label in a multi-task learning configuration? Can pre-training leverage the use of synthetic data to improve the performance? All these questions represent open research directions that could be explored in the future. Recently, researchers have also approached a more human type of learning, known as curriculum learning. This could also be used effectively if, for example, the pre-training and MIL training are distinct stages. Both stages can be iterated multiple times, with hard samples added at each iteration.

Despite the use of attention in some of the articles reviewed, they still incorporate these mechanisms as a type of aggregation layer for their network. However, recent work in natural language processing and in computer vision shows that these mechanisms are considerably more powerful than originally thought^72^. Transformers (neural network architectures based only on attention layers) have shown impressive results. Both Facebook^73^ and Google^74^ have already started exploring these networks for vision and a similar path could also be interesting for CAD systems in pathology.

From a dataset point of view, there is one more issue that has been largely ignored in computational pathology research: a developed model should not be specific to scanning machine output or to particular laboratory configurations. The broad knowledge acquired by the model during the training phase should not be wasted or useless. It is then necessary that the models can either be directly generalised to other scanning machines, or that a small sample of non-annotated WSIs are sufficient to fine-tune a model with similar performance to the original. Thus, broader studies should be conducted in this area, but on a positive note, they do not require large datasets.

In order for these approaches to be used in practice, it is important that researchers develop techniques to inform pathologists about the spatial location that was most responsible for the diagnosis and to explain the reasons for the prediction. Interpretability and explainability have been explored in medical applications of DL^75^, and so they should be present in Computational Pathology use cases^76^, such as CRC diagnosis. The ultimate goal is to create transparent systems that medical professionals can trust and rely on.

## Data Availability

The CRC dataset, from IMP Diagnostics, used in the current study is available on reasonable request through the following email contact: cadpath.ai@impdiagnostics.com. The external PCa dataset was obtained from the Prostate cANcer graDe Assessment (PANDA) challenge and is publicly available through the competition website: https://www.kaggle.com/c/prostate-cancer-grade-assessment.
